# Interferon-Independent Restriction of RNA Virus Entry and Replication by a Class of Damage-Associated Molecular Patterns

**DOI:** 10.1128/mBio.00584-21

**Published:** 2021-04-13

**Authors:** Michael J. Ernandes, Jonathan C. Kagan

**Affiliations:** a Harvard Medical School and Division of Gastroenterology, Boston Children’s Hospital, Boston, Massachusetts, USA; Institut Pasteur

**Keywords:** DAMP, SARS, innate immunity, oxPAPC, virus

## Abstract

Mammalian cells detect microbial molecules known as pathogen-associated molecular patterns (PAMPs) as indicators of potential infection. Upon PAMP detection, diverse defensive responses are induced by the host, including those that promote inflammation and cell-intrinsic antimicrobial activities. Host-encoded molecules released from dying or damaged cells, known as damage-associated molecular patterns (DAMPs), also induce defensive responses. Both DAMPs and PAMPs are recognized for their inflammatory potential, but only the latter are well established to stimulate cell-intrinsic host defense. Here, we report a class of DAMPs that engender an antiviral state in human epithelial cells. These DAMPs include oxPAPC (oxidized 1-palmitoyl-2-arachidonoyl-*sn*-glycero-3-phosphocholine), PGPC (1-palmitoyl-2-glutaryl phosphatidylcholine), and POVPC [1-palmitoyl-2-(5-oxovaleroyl)-*sn*-glycero-3-phosphatidylcholine], oxidized lipids that are naturally released from dead or dying cells. Exposing cells to these DAMPs prior to vesicular stomatitis virus (VSV) infection limits viral replication. Mechanistically, these DAMPs prevent viral entry, thereby limiting the percentage of cells that are productively infected and consequently restricting viral load. We found that the antiviral actions of oxidized lipids are distinct from those mediated by the PAMP Poly I:C, in that the former induces a more rapid antiviral response without the induction of the interferon response. These data support a model whereby interferon-independent defensive activities can be induced by DAMPs, which may limit viral replication before PAMP-mediated interferon responses are induced. This antiviral activity may impact viruses that disrupt interferon responses in the oxygenated environment of the lung, such as influenza virus and SARS-CoV-2.

## Abstract (precis)

In this work, we explored how a class of oxidized lipids, spontaneously created during tissue damage and unprogrammed cell lysis, block the earliest events in RNA virus infection in the human epithelium. This gives us novel insight into the ways that we view infection models, unveiling a built-in mechanism to slow viral growth that neither engages the interferon response nor is subject to known viral antagonism.

## INTRODUCTION

Viral infections represent a major public health burden and a significant source of morbidity and mortality (1). The innate immune system is critical in preventing the establishment of infections. Much of our knowledge of innate immune system activity derives from studies of how the host-encoded proteins known as pattern recognition receptors (PRRs) detect and respond to microbial pathogen-associated molecular patterns (PAMPs) (2). PAMPs are molecular moieties found among potential pathogens, such as bacteria, viruses, and fungi, but not found commonly within healthy eukaryotic cells (3). Cells respond to PAMPs in diverse manners, which can promote cell-extrinsic defensive responses, inflammation, and adaptive immunity. PAMPs also stimulate cell-intrinsic defense mechanisms by altering protein synthesis, altering metabolic activities, and producing interferons (IFNs) which can disrupt critical viral processes such as entry (4), genome replication and transcription (5), translation of viral mRNAs (6), and viral egress (7).

While PAMPs act as an indicator of potential infections, host-encoded molecules can also indicate threats to the host. Host-encoded indicators of potential infection or injury can be released from damaged cells. These molecules are known as damage-associated molecular patterns (DAMPs) (8). An increasingly studied class of DAMPs is represented by oxidized variants of 1-palmitoyl-2-arachidonoyl-*sn*-glycero-3-phosphocholine (PAPC). PAPC is a major component of mammalian cell membranes and becomes oxidized by reactive oxygen species (ROS) released from dead or dying cells. PAPC can be oxidized into numerous derivative chemicals, forming a heterogenous mixture known as oxPAPC (9). The spontaneous formation of oxPAPC has been observed in a variety of contexts of tissue damage or infection, such as acute lung injury and infections with influenza virus and SARS-CoV-1 (10, 11). In these contexts, oxPAPC induces potent inflammatory activities, which mirror their inflammatory activities in other regions of the body, such as within atherosclerotic heart tissue (10, 12, 13).

Within the oxPAPC mixture are specific component lipids that display inflammatory activity. These components are 1-palmitoyl-2-glutaryl phosphatidylcholine (PGPC) and 1-palmitoyl-2-(5-oxovaleroyl)-*sn*-glycero-3-phosphatidylcholine (POVPC). Within macrophages and dendritic cells, PGPC and POVPC have the capacity to induce a newly defined state of phagocyte activation known as hyperactivation, which is associated with inflammasome activities within living cells (14, 15). Thus, oxPAPC and its component lipids display activities that are similar to PAMPs, as representatives from both classes of molecules display inflammatory activities.

DAMPs other than oxPAPC also display inflammatory activities, such as members of the HMG and IL-1 families, and extracellular pools of ATP (16, 17). Despite this common ability of DAMPs to drive inflammation, several gaps exist in our understanding of the symmetry between DAMP and PAMP biology. For example, while PAMPs are recognized for their ability to stimulate an antiviral state of mammalian cells, only IL-1 has been identified as a DAMP with antiviral activity (18, 19). In addition to inducing NF-κB-dependent inflammatory chemokine and cytokine expression, IL-1 induces a signaling pathway that promotes the expression of antiviral genes. This antiviral IL-1 pathway is dependent on the IFN-associated transcription factors IRF1 and STAT1 (18). Here, we report that oxPAPC and its component lipids PGPC and POVPC display antiviral activities. These lipids have the capacity to restrict virus replication in a manner that is not associated with changes in IFN activities. Rather, this activity is associated with an ability to prevent entry of infectious virions into host cells. We found that the oxidized lipid-mediated block in viral entry acts rapidly—faster than the actions of the PAMP Poly I:C. These data support a model whereby select DAMPs and PAMPs may provide overlapping but kinetically and functionally distinct mechanisms of antiviral immunity.

## RESULTS

### oxPAPC and its component lipids restrict VSV replication.

To determine if DAMPs with established inflammatory activities would influence viral infection and replication, we examined the effects of oxPAPC and its component lipids on viral replication in human A549 epithelial cells. A549 cells are commonly used as model epithelia to study PAMP-PRR interactions during infections, yet the influence of DAMPs on this well-characterized cellular system is less well defined. Infections were performed with vesicular stomatitis virus (VSV), which was selected because this pathogen infects epithelia and is often used as a model to study innate immune responses to infection.

**FIG 1:**
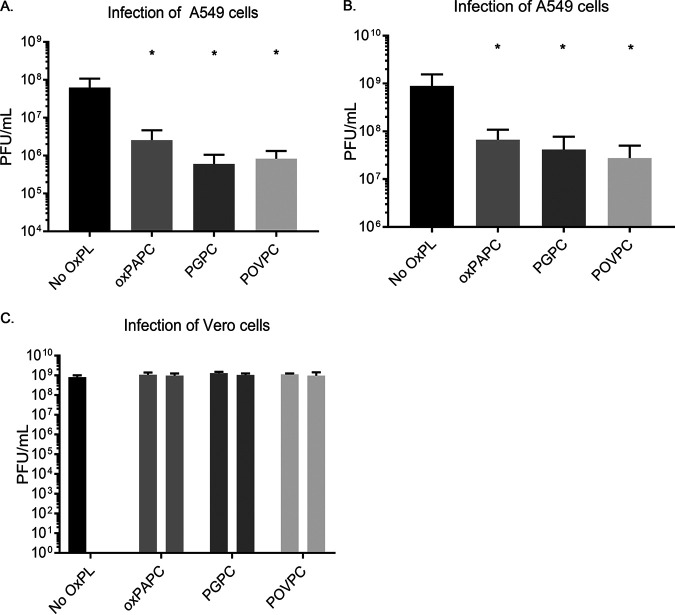
Oxidized phospholipids render cells restrictive to viral infection. (A and B) A549 cells were pretreated with oxPAPC, PGPC, or POVPC at 100 μg/ml for 1 h, followed by infection with VSV at an MOI of 0.1. Supernatants were collected at 8 h postinfection (hpi) (A) for plaque assays or 24 hpi (B). (C) Vero cells were pretreated with oxPAPC, PGPC, or POVPC at 100 μg/ml for 1 h, followed by infection with VSV at an MOI of 0.1. For oxPAPC, PGPC, and POVPC conditions, left-hand bars indicate freshly mixed virus and medium on cells that were pretreated for 1 h with oxidized phospholipids, and right-hand bars indicate virus complexed with oxidized phospholipids for 1 h prior to infection on nonpretreated cells. Statistical analysis was performed using Student’s *t* test, and data shown are representative of 3 biological replicates. *, *P* < 0.05, compared to cells untreated with oxidized phospholipids.

We initiated our studies by treating A549 cells with the heterogenous oxPAPC mixture or the purified components PGPC and POVPC. The concentration of the lipids used (100 μg/ml) corresponds to those found within inflamed tissues and corresponds to concentrations that were established for inflammatory activity in macrophages and dendritic cells (20, 21). One hour after lipid treatment, cells were infected with VSV at a multiplicity of infection (MOI) of 0.1. Viral replication was then monitored by enumerating viral PFU 8 h and 24 h later. At both time points examined, DAMP-treated cells were more restrictive for VSV replication than untreated cells. Specifically, at 8 h postinfection, we observed a 10- to 100-fold reduction in PFU recovered from cells that were treated with oxPAPC, PGPC, or POVPC, compared to untreated cells (Fig. 1A). This restrictive phenotype was also observed at 24 h postinfection, although the magnitude of restriction observed was reduced compared to 8 h (Fig. 1B).

One possible explanation for the defective viral replication observed is that the oxidized lipids may disrupt the enveloped viral particle, rendering them noninfectious. If this possibility is correct, then infection of any cell type should yield results similar to those obtained in A549 cells. We therefore performed experiments similar to those described above in Vero cells, which are commonly used to determine the titer of VSV. Notably, the DAMPs examined had no ability to restrict VSV replication in Vero cells. We confirmed this finding by an extended, 1-h treatment of VSV with the oxidized phospholipids prior to infection and observed no alteration of apparent viral titer (Fig. 1C). These results eliminate the possibility that the oxidized lipids were disrupting the intrinsic infectious potential of VSV. A cell-based activity of oxidized lipids therefore likely explains their antiviral activity.

**FIG 2:**
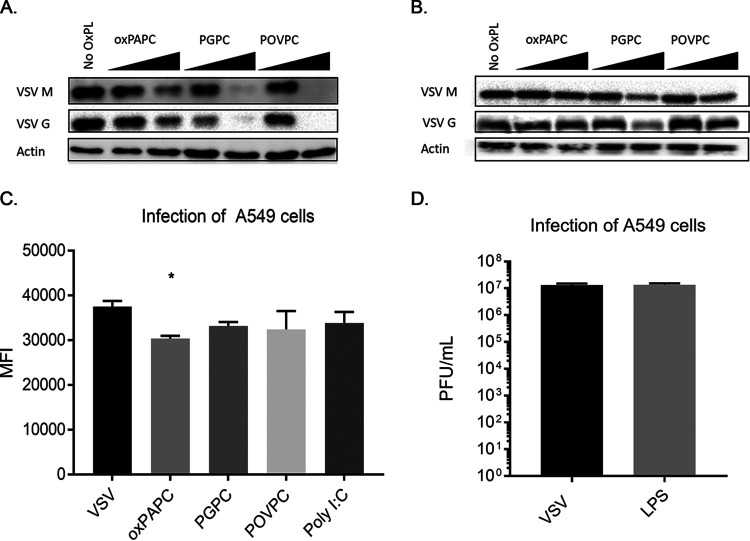
Oxidized phospholipids block VSV protein production in infected cells. (A and B) A549 cells were pretreated with oxPAPC, PGPC, or POVPC at 100 μg/ml for 1 h, followed by infection with VSV at an MOI of 0.1. Lysates were collected at 8 hpi (A) or 24 hpi (B) for Western analysis. (C) A549 cells were pretreated with oxPAPC, PGPC, or POVPC at 100 μg/ml for 1 h, followed by infection with VSV-GFP at an MOI of 0.1 for 8 h. Infected, live cells were determined by distinguishing single cells by forward and side scatter, excluding dead cells, and gating on GFP-positive cells. MFI data were collected from living, infected single cells. (D) A549 cells were pretreated with LPS at 1 μg/ml for 1 h, followed by infection with VSV at an MOI of 0.1. Supernatants were collected at 8 hpi for plaque assays. Statistical analysis was performed using Student’s *t* test, and data shown are representative of 3 biological replicates. *, *P* < 0.05, compared to cells untreated with oxidized phospholipids.

To further explore the restriction of viral replication, we examined the abundance of viral proteins in lysates of cells that were infected in the presence or absence of oxPAPC, PGPC, or POVPC. Consistent with our enumeration of viral PFU, these DAMPs inhibited the production of the viral proteins VSV-G and VSV-M, in a dose-dependent manner (Fig. 2A). Similar to the PFU assays performed, the restrictive phenotypes of the DAMPs examined were most notable 8 h postinfection, with less restriction observed at 24 h postinfection (Fig. 2B). As Western blot assays measure the bulk protein levels within the population, we sought to distinguish whether the differences in viral proteins detected were due to the fewer total cells being infected or to a similar number of infected cells producing less protein. Toward this end, we used a VSV strain that expresses enhanced green fluorescent protein (eGFP) as a reporter for viral replication and protein production. We measured the mean fluorescence intensity (MFI) of cells infected with a VSV strain that expresses GFP (22). In oxidized phospholipid-pretreated cells, we found only a minor decrease in GFP expression on a per-cell basis upon treatment with oxPAPC. No differences in GFP expression were observed upon treatments with PGPC or POVPC (Fig. 2C), suggesting that infected cells are capable of viral protein production.

The higher apparent antiviral potency of PGPC and POVPC, compared to heterogenous oxPAPC, is similar to findings related to their inflammatory activities in macrophages and dendritic cells (15). In these phagocytes, oxPAPC is a less potent stimulator of inflammasome activities than PGPC and POVPC. Mechanistic analysis revealed that the inflammatory activities of these DAMPs depend on the actions of the lipopolysaccharide (LPS) receptor CD14 (15). Indeed, some studies have demonstrated the ability of oxPAPC to stimulate signaling pathways similar to those activated by CD14 and its downstream receptor TLR4 (11). These observations were initially made in the lung, which is the source of the airway epithelial A549 cells used in this study (10). We reasoned that if the antiviral activity of oxPAPC and its component lipids was due to their ability to engage the aforementioned LPS receptors, then LPS treatments should mimic the antiviral activity of oxPAPC. Contrary to this prediction, we found that LPS-treated A549 cells were as permissive for VSV replication as untreated cells (Fig. 2D). Overall, these findings suggest that the DAMPs in question restrict viral replication by a process that does not involve disruption of the virion directly and is not through the process of LPS mimicry.

### oxPAPC and select component lipids prevent VSV-induced cytotoxicity.

**FIG 3:**
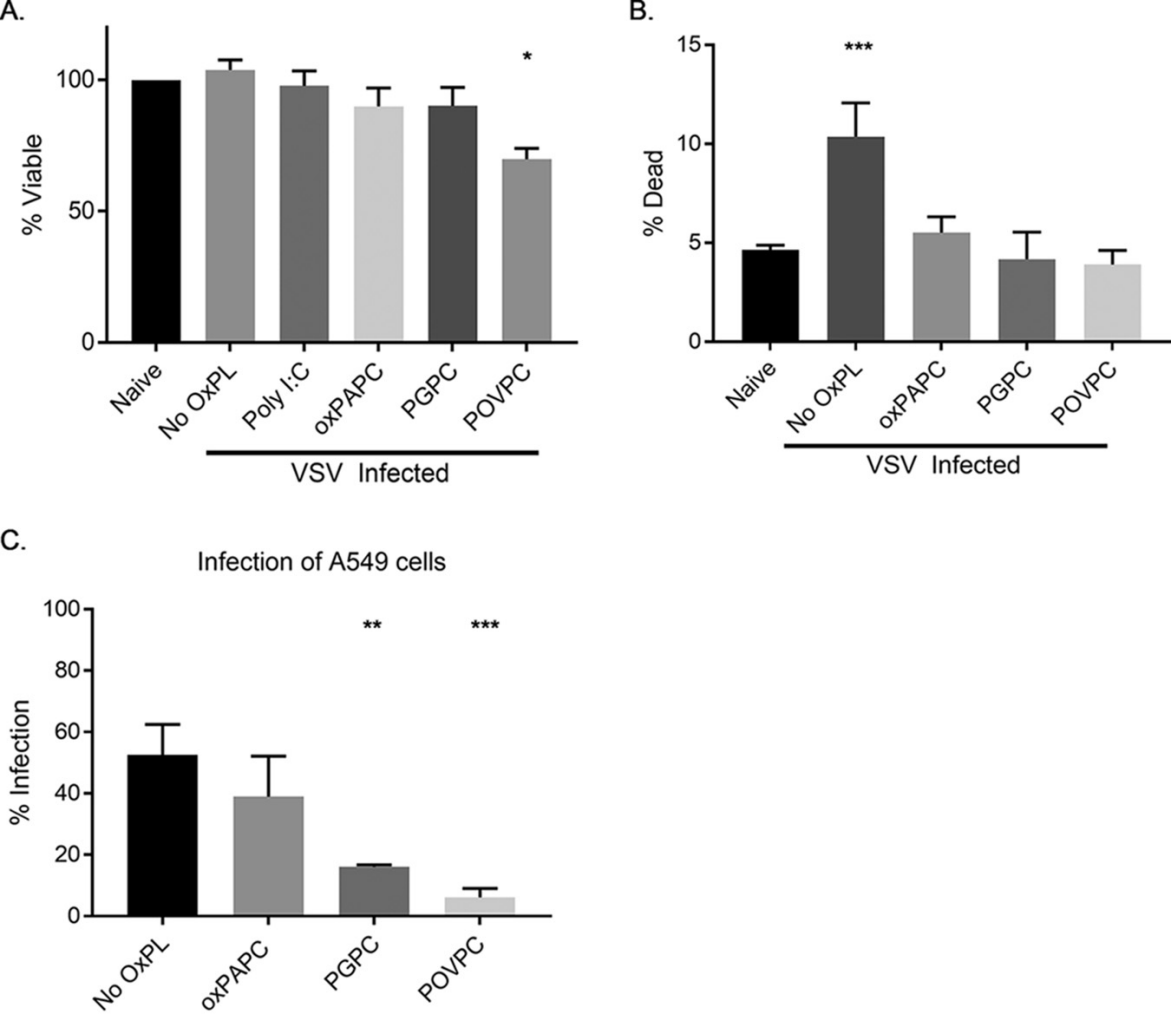
Oxidized phospholipids prevent virus-induced cell death and prevent productive infection. A549 cells were pretreated with Poly I:C at 1 μg/ml or oxidized phospholipids at 100 μg/ml for 1 h, followed by infection with VSV, where indicated. (A) Cells were infected at an MOI of 0.1 and lysed, ATP content was measured using CellTiter-Glo. (B) Cells were infected at an MOI of 1.0, lifted at 6 hpi, and stained with Live/Dead violet amine reactive dye prior to fixation. The dead cell population was identified by forward and side scatter, followed by gating on cells with incorporated dye. (C) Cells were infected using VSV-GFP at an MOI of 1.0, lifted at 6 hpi, and stained with Live/Dead violet amine reactive dye prior to fixation. The infected cell population was identified by forward and side scatter, followed by exclusion of dead cells. Infected cells were determined by GFP positivity. Statistical analysis was performed using Student’s *t* test, and data shown are representative of 3 biological replicates. *, *P* < 0.05; **, *P* < 0.01; ***, *P* < 0.001, compared to cells untreated with oxidized phospholipids.

A common strategy to limit viral infection in cells is the induction of cell death, including through DAMP-induced pyroptosis (23). To determine if oxidized phospholipid treatment was impacting cell viability during viral replication, we performed Western analysis to determine the protein contents within infected cells. None of the oxidized phospholipids examined caused a substantial change on actin abundance at time points when viral replication was prevented (Fig. 2A and B), suggesting cells remained viable. To more directly assess cell viability, we compared the abundance of ATP in uninfected cells to that in cells infected in the presence or absence of oxPAPC, PGPC, or POVPC. Eight-hour infections, which mirror the conditions used to monitor VSV replication (Fig. 1), revealed that only POVPC negatively impacted ATP content of the cells (Fig. 3A). oxPAPC- and PGPC-treated cells displayed no defects in ATP content.

To complement these population-based measures of cell viability, single-cell analysis was performed to determine if the viral restriction is a consequence of the infected cells dying before the virus can replicate. Toward this end, we infected cells as described previously, with and without DAMP treatment. Six hours post infection, we stained the cells with a fixable LIVE/DEAD viability dye. This dye binds to exposed primary amines and consequently acts as a reporter for membrane integrity. Flow cytometry was then used to identify individual viable or dead cells. Using this single-cell assay, we found that VSV infection resulted in an increased detection of dead cells. Interestingly, the VSV-induced increase in dead cell detection was suppressed when infections were performed in the presence of oxPAPC, PGPC, or POVPC (Fig. 3B). This finding is consistent with these DAMPs restricting viral replication, as productive VSV infections often lead to cytopathic effects (24). These collective results eliminate the possibility that oxidized lipids disrupt the viral particle or kill host cells. Rather, at a single-cell level, the cytopathic effects of VSV are suppressed by these DAMPs. oxPAPC and its component lipids may therefore restrict VSV replication by influencing some aspect of the infectious cycle.

### Select oxPAPC component lipids diminish the frequency of cells infected with VSV.

The high sensitivity of our flow cytometric single-cell analysis of cell death prompted us to use a similar approach to further examine the antiviral activities of DAMPs. Toward this end, we infected A549 cells with VSV-GFP, using the percentage of GFP-expressing cells as a measure of percentage of infected cells (22). Infections were performed in the presence or absence of oxPAPC, PGPC, or POVPC, and we both assessed the live/dead signal (described above) and used virus-encoded GFP to identify infected cells (Fig. 3C). This stratagem allowed us to quantify the percentage of infected, living cells. This analysis demonstrated that PGPC and POVPC diminished the number of living cells that were infected with VSV, compared to non-DAMP-treated cells. oxPAPC had less of an impact than its pure component lipids on preventing productive infections of viable cells. The phenotypes observed with these single-cell analyses suggest that oxidized lipids can prevent early events in the infectious process.

### DAMP-mediated antiviral activity is not associated with an induction or alteration of the IFN response.

**FIG 4:**
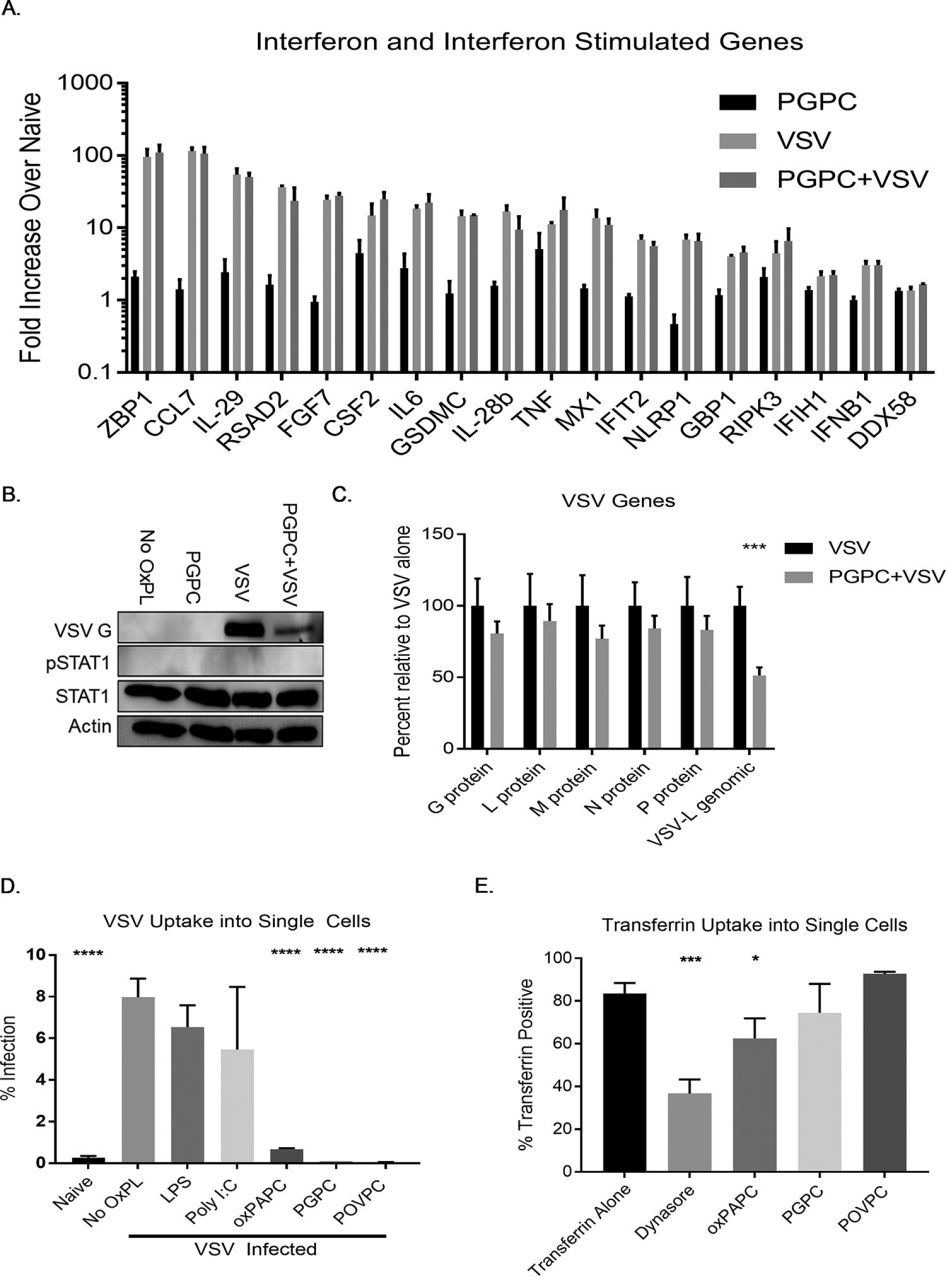
PGPC prevents VSV entry. (A) A549 cells were pretreated with PGPC at 100 μg/ml for 1 h, followed by infection with VSV at an MOI of 0.1. RNA was collected at 3 hpi and purified using the PureLink RNA minikit. nCounter analysis was performed, and fold change was calculated compared to untreated cells. (B) Lysates were collected at 6 hpi for Western analysis. (C) RNA was collected as in panel A, and viral genes were assessed. Comparisons were made between VSV-infected cells with and without PGPC pretreatment. (D) Cells were pretreated for 1 h with LPS or Poly I:C at 1 μg/ml or oxPAPC, PGPC, or POVPC at 100 μg/ml before infection with VSV labeled with Alexa Fluor-594 at an MOI of 0.1 for 1 h, lifted at 6 hpi, and stained with Live/Dead violet amine reactive dye prior to fixation. Infected, live cells were determined by distinguishing single cells by forward and side scatter, excluding dead cells, and gating on Alexa Fluor-594-positive cells. (E) Cells were serum starved with serum-free RPMI, followed by treatment with 80 μM Dynasore, LPS or Poly I:C at 1 μg/ml, or oxidized phospholipids at 100 μg/ml in serum-free RPMI for 1 h. Cells were then lifted and chilled on wet ice. Cells were resuspended in ice-cold PBS with 50 μM fluorescently labeled transferrin and incubated at 37°C for 10 min. Surface-bound transferrin was quenched, and cells were resuspended in ice-cold buffer for flow cytometry. Transferrin-positive cells were determined by distinguishing single cells by forward and side scatter followed by gating on the florescent transferrin signal. Comparisons were made between VSV- or transferrin-positive cells and the other treatments. Statistical analysis was performed using Student’s *t* test, and data shown are representative of 3 biological replicates. *, *P* < 0.05; ***, *P* < 0.001; ****, *P* < 0.0001.

To further elucidate the mechanism by which viral replication is restricted by DAMPs, we quantified the expression of select immune genes and all transcripts encoded by VSV, including its genomic RNA. We used PGPC as a representative DAMP based on its ability to restrict VSV replication without impact on cell viability. RNA was collected from infected cells and analyzed by nCounter analysis, which is a microarray-like procedure that directly quantifies RNA within a sample. We chose a 3-h infection time, as this point represents a time where only a single round of viral replication will have been completed (25). From our nCounter analysis on immune genes, several patterns emerged (Fig. 4A). The type III IFNs (IL-28b and IL-29) and several IFN-stimulated genes (ISGs) were expressed in response to infection. Included among the VSV-induced ISGs are MX1, RSAD2, ZBP1, IFIH1, and DDX58, which represent critical effectors and sensors of the antiviral response (26). Notably, PGPC treatment had a minimal impact on the expression of these IFNs and ISGs, and PGPC had no impact on the expression of these genes within infected cells.

We noted a few factors, such as tumor necrosis factor alpha (TNF-α), IL-6, and CSF2, which were upregulated by PGPC alone, but there was no difference in transcript abundance when comparing VSV infection with or without PGPC (Fig. 4A). Finally, we noted that the expression of the prototype type I IFN, IFNB1, was induced modestly by VSV infection. The minimal expression of IFNB1 and the known ability of VSV to interfere with IFNB1 translation promoted us to examine IFN activity within infected cells (27). If a pool of IFNB1 was secreted from cells, then the IFN-responsive transcription factor STAT1 should be phosphorylated. Western analysis of infected cells demonstrated a lack of STAT1 phosphorylation as late as 6 h postinfection (Fig. 4B). The presence of PGPC during infection diminished the production of VSV-G proteins but did not impact the lack of STAT1 phosphorylation observed. Collectively, these data indicate that PGPC has no ability to stimulate IFN expression or ISG expression or impact the IFN and ISG activities present within infected cells. Based on these findings, we suggest an IFN-independent mechanism of DAMP-mediated antiviral activity.

### oxPAPC and component lipids restrict viral entry.

nCounter analysis allowed for the quantification of VSV RNA in the same cells that we examined for immune gene expression. As expected, no VSV RNA was found in uninfected cells and high levels were detected within infected cells. The presence of PGPC did not impact the abundance of RNAs encoding the viral proteins L, M, N, G, and P. In contrast, PGPC-treated cells displayed a decrease in the abundance of viral genomic RNA (Fig. 4C). This observed difference in genomic viral RNA abundance suggests diminished entry of virions. To determine the impact of DAMPs on viral entry, we labeled VSV with the fluorophore Alexa Fluor-594, a bright dye that is compatible with single-cell flow cytometric analysis. Labeled virus was then used to infect A549 cells in the presence or absence of oxPAPC, PGPC, or POVPC. After 1 h of DAMP pretreatment, cells were infected for 1 h and were treated with trypsin to remove all cell surface extracellular virus. Thus, all cell-associated fluorescence should derive from virions that entered the cell. Notably, we observed that DAMP-treated cells contained less virus than cells that were not treated with any oxidized lipids. Depending on the lipid examined, we detected a 12- to 200-fold decrease in the amount of viral entry into DAMP-treated cells (Fig. 4D).

In contrast to the inhibitory effects on VSV entry, the entry of fluorescent transferrin into cells was either unaffected (PGPC and POVPC) or minimally affected (oxPAPC) by DAMP treatment (Fig. 4E). As expected, the dynamin inhibitor Dynasore interfered with the entry of transferrin into cells. These observations, taken together, show that the DAMP-mediated viral replication is limited via restriction of viral entry.

### DAMP-mediated antiviral responses occur with faster kinetics than those mediated by PAMPs.

**FIG 5:**
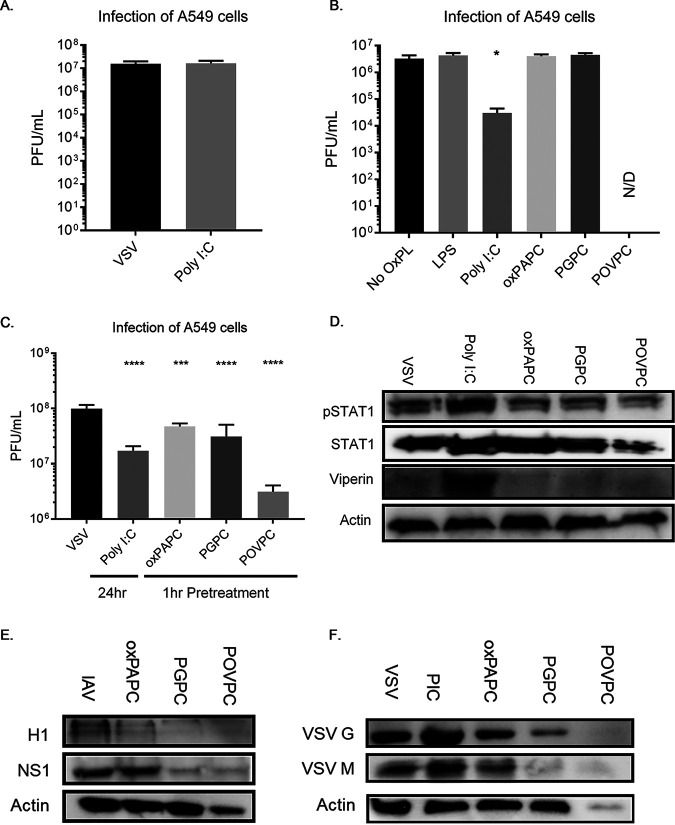
Oxidized phospholipid-mediated restriction is kinetically distinct from interferon-mediated restriction and is limited by neither cell type nor virus. (A) A549 cells were pretreated with Poly I:C at 5 μg/ml for 1 h, followed by infection with VSV. Supernatants were collected at 8 hpi for plaque assays. (B) A549 cells were pretreated with oxPAPC, PGPC, or POVPC at 100 μg/ml; Poly I:C at 5 μg/ml; or LPS at 1 μg/ml for 24 h, followed by infection with VSV. Supernatants were collected at 8 hpi for plaque assays. (C) A549 cells were pretreated with oxPAPC, PGPC, or POVPC at 100 μg/ml for 1 h or Poly I:C at 5 μg/ml for 24 h, followed by infection with VSV. Supernatants were collected at 8 hpi for plaque assays. (D) Lysates were collected for Western analysis. (E) A549 cells were pretreated with oxPAPC, PGPC, or POVPC at 100 μg/ml for 1 h, followed by infection with influenza A virus (IAV) at an MOI of 0.1. Lysates were collected after 12 h for Western analysis. (F) NOK cells were pretreated with oxPAPC, PGPC, or POVPC at 100 μg/ml or Poly I:C at 5 μg/ml for 1 h, followed by infection with VSV at an MOI of 0.1. Lysates were collected after 8 h for Western analysis. Statistical analysis was performed using Student’s *t* test, and data shown are representative of 3 biological replicates. *, *P* < 0.05; ***, *P* < 0.001; ****, *P* < 0.0001, compared to cells untreated with oxidized phospholipids.

The findings reported thus far suggest that the DAMPs examined display a rapid IFN-independent activity that prevents VSV entry and replication. These activities are distinct from those typically discussed for PAMPs, where antiviral activities often take hours to manifest and are mediated by IFNs (28). Consistent with this idea, while we found throughout this study that a 1-h pretreatment of cells with oxidized phospholipid DAMPs is sufficient to prevent VSV replication, 1-h pretreatments with the PAMP Poly I:C did not (Fig. 5A). In addition, we found that Poly I:C had no significant impact on VSV entry into cells, whereas the DAMPs examined suppressed VSV entry (Fig. 4D). In contrast to the lack of rapid antiviral activities of Poly I:C, treatments of cells for 24 h with Poly I:C prior to infection strongly suppressed VSV replication. Interestingly, 24-h pretreatments with oxPAPC, PGPC, or POVPC did not limit viral replication, though POVPC appeared to be acutely toxic in these conditions (Fig. 5B). The functional and kinetic differences observed when comparing Poly I:C to oxidized lipids suggest that DAMPs and PAMPs provide distinct but complementary benefits to host antiviral immunity. To compare the relative antiviral effects of DAMPs and Poly I:C side-by-side, we pretreated cells for 1 h with the oxidized phospholipids and for 24 h with Poly I:C before infecting at a low MOI to assess viral replication. We found a similar degree of viral restriction under these conditions (Fig. 5C) and further confirmed the IFN-mediated nature of Poly I:C-mediated restriction via Western analysis, noting the presence of phosphorylated STAT1 and viperin (Fig. 5D). DAMPs may act early, in an IFN-independent manner, to restrict viral entry, while PAMPs can act with delayed kinetics to induce IFNs and provide long-term protection.

### DAMP-mediated antiviral responses extend to other cell types and other viral infection systems.

To explore the generalizability of the DAMP-mediated viral restriction, we tested our A549 infection system using influenza A/PR8/1934 virus. By Western analysis, we found that pretreatment with the oxidized phospholipids diminished the level of viral proteins in a manner similar to VSV infection (Fig. 5E). In an analogous experiment, we used normal oral keratinocytes (NOKs), another cell type that is likely to be exposed to high oxygen content and is abundant in a tissue (skin) that is the natural portal of VSV entry after an insect bite (29). These cells were pretreated with Poly I:C or DAMPs for 1 h followed by infection with VSV, where we again found a diminution of viral proteins from DAMP exposure, indicating restriction of VSV infection in NOKs (Fig. 5F).

## DISCUSSION

Slowing the progress of infections within the host is one of the critical functions of the innate immune system. Classically, pathogens stimulate PRRs, which assemble supramolecular organizing complexes (SMOCs) to coordinate a variety of inflammatory and antiviral responses (30). To undermine these responses and establish a replicative niche, viral pathogens actively disrupt innate immune signal transduction, immune gene transcription and translation, and other critical antiviral host processes (31).

Alternative methods, less reliant on PRR-dependent transcriptional responses, appear necessary to combat these immune evasion strategies. Cryptic innate immune mechanisms must exist to fill in and supplement the gaps when well-known innate immune mechanisms are insufficient. Preventing viral entry represents the earliest opportunity to prevent infection; paradoxically, examples of innate immune viral restriction by blocking viral entry are scant. Indeed, a recently reported set of studies highlighted the ISG Ly6E as a factor that promotes, rather than inhibits, viral infection, and the SARS-CoV-2 entry factor ACE2 is also induced by the actions of IFNs (32). Due to the profound immune antagonism inherent in viral replication and the independent sensing of PAMPs and DAMPs, DAMPs provide an end-run around conventional viral strategies to antagonize the innate immune system. Our discovery of a cellular response to DAMPs that restricts viral entry represents a useful strategy to prevent viral replication prior to the time when IFNs are induced. The specific host factor that determines DAMP-mediated prevention of viral entry and the spectrum of viruses whose entry events are influenced by DAMPs remain important questions for future study.

While the identity of the DAMP-induced antiviral factor(s) remains undefined, the rapid-acting nature of the oxidized lipids examined raises an interesting possible benefit to this defense strategy. This newly identified DAMP-mediated viral restriction should effectively circumvent most viral antagonism strategies, as it is engaged prior to infection. While some secreted immune antagonists exist, the preponderance of viral immune evasion strategies are situated within the cell and are associated with the replicative cycle of the virus (31). oxPAPC and its components PGPC and POVPC, in rendering epithelial cells less susceptible to viral infection, undercut the immune evasive activities of VSV and influenza virus before infection can be established.

It is interesting to consider how these findings inform our conception of infection acquisition. In cases of skin disruption, where underlying tissues become exposed to oxygen before they would potentially encounter viral pathogens (or in any highly oxygenated environment such as the lung epithelium), we would expect the oxidized phospholipids we have examined to be among the first inducers of host defense. The lung epithelium is a major site of interest for viral pathogenesis as the principal site of infection for a variety of respiratory pathogens. Though these cells are not traditionally considered immune cells, as they are not of hematopoietic origin, they are likely to be among the first cell types infected by a variety of pathogens such as coronaviruses (33) and influenza virus (34). By impeding early events in viral infection, these oxidized phospholipids potentially widen the range of time before the cell is overwhelmed by the infectious process. This longer window of time may allow PRRs to activate IFN-mediated antiviral pathways, which likely mediate the most effective antiviral responses. This schema mirrors a traditional view of the innate immune system, which acts early in infection to slow pathogenic growth and spread, allowing the adaptive immune system to engage with the pathogen. Our findings expand the purview of DAMP signaling to include antiviral activities and provide a mandate to more closely examine the roles of DAMPs in protecting vulnerable cells from infection.

## MATERIALS AND METHODS

### Resource availability.

Further information and requests for reagents and resources should be directed to Jonathan Kagan (Jonathan.Kagan@childrens.harvard.edu).

### Material availability.

There are restrictions on the availability of A594-labeled virus due to a scarcity of the reagent. This study did not generate any other unique reagents.

### Experimental model and subject details. (i) Cell culture.

A549 cells, a male adenocarcinoma-derived lung epithelium cell line, were cultured in Roswell Park Memorial Institution 1640 medium (RPMI 1640; Lonza Bioscience, Rockville, MD, USA) supplemented with 10% fetal bovine serum (FBS). These cells were a gift from the DeCaprio lab (Harvard Medical School). RPMI was also supplemented with penicillin-streptomycin (Pen-Strep), l-glutamine, and sodium pyruvate (all from Millipore-Sigma, Burlington, MA, USA) used at 1:100 (vol/vol). This full medium is referred to as “complete” RPMI. Viral infections were performed in a similar medium supplemented with 1% FBS instead of 10% FBS. A549 cells were passaged by lifting with trypsin (0.25%) and 0.1% EDTA (Millipore-Sigma) for 5 min before quenching with complete RPMI, followed by centrifugation to remove residual trypsin. A549 cells were passaged by splitting at a 1:5 ratio from nearly confluent flasks.

Vero cells, derived from female African green monkey kidney cells, were cultured in Dulbecco’s modified Eagle’s medium (DMEM; Sigma) supplemented with Pen-Strep, l-glutamine, and sodium pyruvate at 1:100 with 10% FBS (for complete DMEM) or 1% FBS for viral infections (for vDMEM). These cells were passaged by lifting with trypsin (0.25%) and 0.1% EDTA (Millipore-Sigma) for 5 min before quenching with complete DMEM, followed by centrifugation to remove residual trypsin. Vero cells were passaged by splitting at a 1:5 ratio from nearly confluent flasks. Normal oral keratinocytes (NOKs) were immortalized with human telomerase reverse transcriptase (hTERT) and were cultured in keratinocyte–serum-free medium supplemented with the provided cell culture supplements (Thermo Fisher, Waltham, MA, USA). These cells were passaged by lifting with trypsin (0.25%) and 0.1% EDTA (Millipore-Sigma) for 5 min before quenching with complete DMEM, followed by centrifugation to remove residual trypsin. NOK cells were passaged by splitting at a 1:3 ratio from mostly confluent flasks.

### (ii) Oxidized lipids.

Oxidized 1-palmitoyl-2-arachidonoyl-*sn*-glycero-3-phosphocholine (oxPAPC) (InvivoGen, San Diego, CA) was supplied as a pure solid and was resuspended in RPMI supplemented with 1% FBS, Pen-Strep, l-glutamine, and sodium pyruvate (vRPMI) at a final concentration of 1 mg/ml. 1-Palmitoyl-2-glutaryl phosphatidylcholine (PGPC) and 1-palmitoyl-2-(5-oxovaleroyl)-*sn*-glycero-3-phosphatidylcholine (both from Cayman Chemical, Ann Arbor, MI) were both supplied in a small volume of 100% ethanol at a reported purity of ≥98%. Ethanol was evaporated off in a fume hood using N_2_ gas, followed by immediate resuspension in RPMI supplemented with 1% FBS, Pen-Strep, l-glutamine, and sodium pyruvate (vRPMI) at a final concentration of 1 mg/ml. For transferrin uptake assay, oxidized lipids were resuspended in in RPMI supplemented with Pen-Strep, l-glutamine, and sodium pyruvate (serum-free RPMI).

### (iii) Virus and viral infection.

Indiana strain vesicular stomatitis virus (VSV) and VSV-GFP were generous gifts from the Sean Whelan Laboratory (Harvard Medical School) and propagated in male golden hamster BSRT7 cells. Cells were plated in T75 flasks until 85% confluent and infected at an MOI of 3 in vDMEM for 20 to 24 h, until all cells showed distinct cytopathic effect (CPE). Supernatants were collected and spun down at 2,000 relative centrifugal force (rcf) for 5 min to pellet any floating cells. Cleared supernatants were then ultracentrifuged at 21,000 × *g* for 90 min at 4°C in the Ty 50.2 rotor (Becton Coulter, Brea, CA, USA). The resultant pellet was then resuspended in NTE buffer (10 mM Tris, 100 mM NaCl, 1 mM EDTA, pH 7.4) overnight at 4°C. The resuspended virus was then further purified via sucrose cushion purification in a 10% sucrose solution via ultracentrifuging using the SW50.1 rotor at 41,000 rpm for 60 min at 4°C. This was resuspended in NTE buffer overnight at 4°C and aliquoted into 10-μl aliquots. Alexa 594-labeled virus was created in-house by using the Alexa Fluor-594 *N*-hydroxysuccinimide (NHS) ester (succinimidyl ester) (Thermo Fisher, Waltham, MA, USA) kit on highly purified virus following the manufacturer’s procedure. The resultant virus went through an additional sucrose gradient purification to ensure specificity in the assays.

Influenza A/PR8/1934 virus was a generous gift from the Daniel Lingwood Lab (Harvard University) and propagated in Madin-Darby canine kidney (MDCK) cells. Confluent T-25 flasks were infected with PR8 at a 1:100 ratio for 1 h, followed by the addition of influenza virus growth medium, and incubated until at least 75% of the cells showed CPE. Influenza virus growth medium is made using DMEM supplemented with 7.5% bovine serum albumin (BSA) (Sigma-Aldrich) to a final concentration of 0.2%, Pen-Strep, l-glutamine, 25 mM HEPES, and 0.01% TPCK (tosylsulfonyl phenylalanyl chloromethyl ketone)-treated trypsin (Sigma-Aldrich). Virus-laden medium is then harvested and spun at 500 × *g* for 15 min to clear cell debris. Virus was then concentrated using an Amicon filter with a 30-kDa cutoff (Millipore Sigma) and titrated by plaque assay and hemagglutination assay.

Infections of NOK and A549 cells were performed as follows for protein and RNA purification, plaque assay, ATP measurement, and flow cytometric analysis. Cells were plated the previous evening to allow proper adhesion to the plate, always plating at least one unneeded well for an accurate count of cells in the well to calculate multiplicity of infection (MOI). Prior to viral infections, cells were pretreated with oxidized phospholipids, LPS, or Poly I:C at indicated concentrations in culturing medium for 1 h in 5% CO_2_ at 37°C. This medium was aspirated and replaced with vRPMI containing virus at a specified MOI and/or oxidized phospholipid for 1 h in 5% CO_2_ at 37°C to allow attachment and internalization of the viral particles. After 1 h, the virus-laden medium was removed and replaced with culturing medium including oxidized phospholipids, as indicated. After the indicated times of infection, supernatants were collected to quantify infectious virus and the attached cells were either collected for flow cytometry or lysed for downstream RNA, protein, and metabolite analysis.

### (iv) Plaque assays.

Vero cells were plated (5 × 10^5^) in a 12-well plate in complete DMEM to form a confluent monolayer. Culture medium was aspirated, and supernatants from previous samples were added in 100-μl increments after 10-fold dilution series in vDMEM, in duplicate. Virus-laden medium was applied to cells for 1 h to allow adhesion and entrance into the susceptible cells. After 1 h, virus-laden supernatants were aspirated and agarose overlay was applied. After 20 to 24 h, the agarose plug was removed and the monolayer was fixed using a 10% ethanol, 0.05% crystal violet solution. The plaques were enumerated, and the initial concentration of infectious virus could then be back-calculated.

### (v) Western analysis.

A549 cells (0.4 × 10^6^) or NOK cells (0.2 × 10^6^) were seeded in 12-well plates, stimulated as previously described, and subsequently lysed in 300 μl 1× Laemmli buffer. The samples were incubated at 65°C for 15 min and then passed through a 26-gauge needle using a 1-ml BD syringe (Becton Dickinson, Franklin Lakes, NJ, USA). Fifteen microliters of this lysate was then separated by SDS-PAGE followed by Western analysis.

### (vi) nCounter analysis.

A549 cells (0.4 × 10^6^) were seeded in 12-well plates, stimulated as described above, and subsequently lysed following the directions for the PureLink RNA mini-purification kit (Thermo Fisher), preceded by use of QIAshredder (Qiagen, Hilden, Germany) to clear the lysate. After RNA purification, samples were standardized to 20 μg/μl and run using the nCounter Sprint profiler (NanoString, Seattle, WA, USA). Data were collated using nSolver4.0 (NanoString) and analyzed using GraphPad Prism 7 (GraphPad, San Diego, CA, USA).

### (vii) Transferrin uptake assay.

A549 cells were plated (0.8 × 10^6^) in 6-well plates and allowed to set and grow overnight. Cells were starved in serum-free DMEM for 1 h. The cells were subsequently lifted using 5 mM EDTA in phosphate-buffered saline (PBS) and moved to 5-ml round-bottom polystyrene tubes. Cells were then incubated on wet ice for 1 h in oxidized phospholipids, LPS, or 80 μM Dynasore in serum-free RPMI, followed by a wash step. Cells were then incubated with Alexa 633-transferrin or Alexa 488-transferrin (50 μg/ml) for 10 min on wet ice in cold serum-free RPMI in a 100-μl volume. Cells were then incubated at 37°C for 10 min to allow internalization of the transferrin. After incubation, cells were immediately washed in ice-cold serum-free RPMI, followed by a wash in ice-cold PBS. Remaining cell surface transferrin was quenched by a 4°C acid wash (0.1 M glycine, 150 mM NaCl, pH 3) followed by a PBS wash, followed by additional acid wash. The cells were then resuspended in flow cytometry buffer (PBS supplemented with 2.5 mM EDTA, 1% FBS, Pen-Strep), filtered through a 40-μm cell strainer, and analyzed by flow cytometry on a BD LSRFortessa (Becton Dickinson).

### (viii) Cell viability analysis.

ATP levels were measured using CellTiter-Glo (Promega), a luciferase-based assay for ATP in living cells used as a measure of cell viability. Assays were performed using the manufacturer’s protocol using untreated cells as a positive control as the 100% viable benchmark. Luminescent output was measured using a Tecan Spark plate reader.

### (ix) Flow cytometry analysis.

A549 cells (0.4 × 10^6^) were seeded in 12-well plates and infected as described above. Cells were lifted from the plate using PBS and 2.5 mM EDTA and spun down at 400 × *g* for 5 min at 4°C. Cells were next washed in PBS containing 2.5% EDTA, 1% FBS, and Pen-Strep (flow buffer) and then stained for viability using LIVE/DEAD violet dead cell stain (Invitrogen, Carlsbad, CA) at 1:1,000 dilution in cold PBS for 25 min at 4°C. Subsequently, cells were washed and fixed using BD Cytofix at 1:100 (Becton Dickinson) for 30 min at 4°C. Prior to running the samples on the flow cytometer, cells were filtered through a 40-μm cell strainer.

Flow cytometry data were collected using the BD LSRFortessa (Becton Dickinson). Viability information was assessed from the LIVE/DEAD stain, and infection data were assessed either from the GFP signal resulting from VSV-GFP infection or from the A594 signal in A594-labeled VSV.

## References

[1] Price-Haywood EG, Burton J, Fort D, Seoane L. 2020. Hospitalization and mortality among black patients and white patients with covid-19. N Engl J Med 382:2534–2543. doi:10.1056/NEJMsa2011686. 32459916 PMC7269015

[2] Janeway CA. 1989. Approaching the asymptote? Evolution and revolution in immunology. Cold Spring Harb Symp Quant Biol 54:1–13. doi:10.1101/SQB.1989.054.01.003. 2700931

[3] Takeuchi O, Akira S. 2010. Pattern recognition receptors and inflammation. Cell 140:805–820. doi:10.1016/j.cell.2010.01.022. 20303872

[4] Feeley EM, Sims JS, John SP, Chin CR, Pertel T, Chen L-M, Gaiha GD, Ryan BJ, Donis RO, Elledge SJ, Brass AL. 2011. IFITM3 inhibits influenza A virus infection by preventing cytosolic entry. PLoS Pathog 7:e1002337. doi:10.1371/journal.ppat.1002337. 22046135 PMC3203188

[5] Narvaiza I, Linfesty DC, Greener BN, Hakata Y, Pintel DJ, Logue E, Landau NR, Weitzman MD. 2009. Deaminase-independent inhibition of parvoviruses by the APOBEC3A cytidine deaminase. PLoS Pathog 5:e1000439. doi:10.1371/journal.ppat.1000439. 19461882 PMC2678267

[6] Zhang S, Sun Y, Chen H, Dai Y, Zhan Y, Yu S, Qiu X, Tan L, Song C, Ding C. 2014. Activation of the PKR/eIF2α signaling cascade inhibits replication of Newcastle disease virus. Virol J 11:62. doi:10.1186/1743-422X-11-62. 24684861 PMC3994276

[7] Weidner JM, Jiang D, Pan XB, Chang J, Block TM, Guo JT. 2010. Interferon-induced cell membrane proteins, IFITM3 and tetherin, inhibit vesicular stomatitis virus infection via distinct mechanisms. J Virol 84:12646–12657. doi:10.1128/JVI.01328-10. 20943977 PMC3004348

[8] Yang H, Hreggvidsdottir HS, Palmblad K, Wang H, Ochani M, Li J, Lu B, Chavan S, Rosas-Ballina M, Al-Abed Y, Akira S, Bierhaus A, Erlandsson-Harris H, Andersson U, Tracey KJ. 2010. A critical cysteine is required for HMGB1 binding to Toll-like receptor 4 and activation of macrophage cytokine release. Proc Natl Acad Sci U S A 107:11942–11947. doi:10.1073/pnas.1003893107. 20547845 PMC2900689

[9] Bochkov VN, Oskolkova OV, Birukov KG, Levonen A-L, Binder CJ, Stöckl J. 2010. Generation and biological activities of oxidized phospholipids. Antioxid Redox Signal 12:1009–1059. doi:10.1089/ars.2009.2597. 19686040 PMC3121779

[10] Imai Y, Kuba K, Neely GG, Yaghubian-Malhami R, Perkmann T, Van Loo G, Ermolaeva M, Veldhuizen R, Leung YHC, Wang H, Liu H, Sun Y, Pasparakis M, Kopf M, Mech C, Bavari S, Peiris JSM, Slutsky AS, Akira S, Hultqvist M, Holmdahl R, Nicholls J, Jiang C, Binder CJ, Penninger JM. 2008. Identification of oxidative stress and Toll-like receptor 4 signaling as a key pathway of acute lung injury. Cell 133:235–249. doi:10.1016/j.cell.2008.02.043. 18423196 PMC7112336

[11] Shirey KA, Lai W, Scott AJ, Lipsky M, Mistry P, Pletneva LM, Karp CL, McAlees J, Gioannini TL, Weiss J, Chen WH, Ernst RK, Rossignol DP, Gusovsky F, Blanco JCG, Vogel SN. 2013. The TLR4 antagonist Eritoran protects mice from lethal influenza infection. Nature 497:498–502. doi:10.1038/nature12118. 23636320 PMC3725830

[12] Miller YI, Choi S-H, Wiesner P, Fang L, Harkewicz R, Hartvigsen K, Boullier A, Gonen A, Diehl CJ, Que X, Montano E, Shaw PX, Tsimikas S, Binder CJ, Witztum JL. 2011. Oxidation-specific epitopes are danger-associated molecular patterns recognized by pattern recognition receptors of innate immunity. Circ Res 108:235–248. doi:10.1161/CIRCRESAHA.110.223875. 21252151 PMC3075542

[13] Schuh C, Wimmer I, Hametner S, Haider L, Van Dam A-M, Liblau RS, Smith KJ, Probert L, Binder CJ, Bauer J, Bradl M, Mahad D, Lassmann H. 2014. Oxidative tissue injury in multiple sclerosis is only partly reflected in experimental disease models. Acta Neuropathol 128:247–266. doi:10.1007/s00401-014-1263-5. 24622774 PMC4102830

[14] Zanoni I, Tan Y, Di Gioia M, Broggi A, Ruan J, Shi J, Donado CA, Shao F, Wu H, Springstead JR, Kagan JC. 2016. An endogenous caspase-11 ligand elicits interleukin-1 release from living dendritic cells. Science 352:1232–1236. doi:10.1126/science.aaf3036. 27103670 PMC5111085

[15] Zanoni I, Tan Y, Di Gioia M, Springstead JR, Kagan JC. 2017. By capturing inflammatory lipids released from dying cells, the receptor CD14 induces inflammasome-dependent phagocyte hyperactivation. Immunity 47:697–709.e3. doi:10.1016/j.immuni.2017.09.010. 29045901 PMC5747599

[16] Scaffidi P, Misteli T, Bianchi ME. 2002. Release of chromatin protein HMGB1 by necrotic cells triggers inflammation. Nature 418:191–195. doi:10.1038/nature00858. 12110890

[17] Vénéreau E, Ceriotti C, Bianchi ME. 2015. DAMPs from cell death to new life. Front Immunol 6:422. doi:10.3389/fimmu.2015.00422. 26347745 PMC4539554

[18] Orzalli MH, Smith A, Jurado KA, Iwasaki A, Garlick JA, Kagan JC. 2018. An antiviral branch of the IL-1 signaling pathway restricts immune-evasive virus replication. Mol Cell 71:825–840.e6. doi:10.1016/j.molcel.2018.07.009. 30100266 PMC6411291

[19] Iwata M, Vieira J, Byrne M, Horton H, Torok-Storb B. 1999. Interleukin-1 (IL-1) inhibits growth of cytomegalovirus in human marrow stromal cells: inhibition is reversed upon removal of IL-1. Blood 94:572–578. doi:10.1182/blood.V94.2.572.414k18_572_578. 10397724

[20] Chu LH, Indramohan M, Ratsimandresy RA, Gangopadhyay A, Morris EP, Monack DM, Dorfleutner A, Stehlik C. 2018. The oxidized phospholipid oxPAPC protects from septic shock by targeting the non-canonical inflammasome in macrophages. Nat Commun 9:996. doi:10.1038/s41467-018-03409-3. 29520027 PMC5843631

[21] Di Gioia M, Spreafico R, Springstead JR, Mendelson MM, Joehanes R, Levy D, Zanoni I. 2020. Endogenous oxidized phospholipids reprogram cellular metabolism and boost hyperinflammation. Nat Immunol 21:42–53. doi:10.1038/s41590-019-0539-2. 31768073 PMC6923570

[22] Dalton KP, Rose JK. 2001. Vesicular stomatitis virus glycoprotein containing the entire green fluorescent protein on its cytoplasmic domain is incorporated efficiently into virus particles. Virology 279:414–421. doi:10.1006/viro.2000.0736. 11162797

[23] Upton JW, Chan FK. 2014. Staying alive: cell death in antiviral immunity. Mol Cell 54:273–280. doi:10.1016/j.molcel.2014.01.027. 24766891 PMC4010939

[24] Balachandran S, Porosnicu M, Barber GN. 2001. Oncolytic activity of vesicular stomatitis virus is effective against tumors exhibiting aberrant p53, Ras, or myc function and involves the induction of apoptosis. J Virol 75:3474–3479. doi:10.1128/JVI.75.7.3474-3479.2001. 11238874 PMC114141

[25] Timm A, Yin J. 2012. Kinetics of virus production from single cells. Virology 424:11–17. doi:10.1016/j.virol.2011.12.005. 22222212 PMC3268887

[26] Schoggins JW, Rice CM. 2011. Interferon-stimulated genes and their antiviral effector functions. Curr Opin Virol 1:519–525. doi:10.1016/j.coviro.2011.10.008. 22328912 PMC3274382

[27] Ahmed M, McKenzie MO, Puckett S, Hojnacki M, Poliquin L, Lyles DS. 2003. Ability of the matrix protein of vesicular stomatitis virus to suppress beta interferon gene expression is genetically correlated with the inhibition of host RNA and protein synthesis. J Virol 77:4646–4657. doi:10.1128/jvi.77.8.4646-4657.2003. 12663771 PMC152115

[28] Trapp S, Derby NR, Singer R, Shaw A, Williams VG, Turville SG, Bess JW, Lifson JD, Robbiani M. 2009. Double-stranded RNA analog poly(I:C) inhibits human immunodeficiency virus amplification in dendritic cells via type I interferon-mediated activation of APOBEC3G. J Virol 83:884–895. doi:10.1128/JVI.00023-08. 19004943 PMC2612396

[29] Rozo-Lopez P, Drolet B, Londoño-Renteria B. 2018. Vesicular stomatitis virus transmission: a comparison of incriminated vectors. Insects 9:190. doi:10.3390/insects9040190. PMC6315612 30544935

[30] Tan Y, Kagan JC. 2019. Innate immune signaling organelles display natural and programmable signaling flexibility. Cell 177:384–398.e11. doi:10.1016/j.cell.2019.01.039. 30853218 PMC6710629

[31] Faul EJ, Lyles DS, Schnell MJ. 2009. Interferon response and viral evasion by members of the family Rhabdoviridae. Viruses 1:832–851. doi:10.3390/v1030832. 21994572 PMC3185512

[32] Ziegler CGK, Allon SJ, Nyquist SK, Mbano IM, Miao VN, Tzouanas CN, Cao Y, Yousif AS, Bals J, Hauser BM, Feldman J, Muus C, Wadsworth MH, II, Kazer SW, Hughes TK, Doran B, Gatter GJ, Vukovic M, Taliaferro F, Mead BE, Guo Z, Wang JP, Gras D, Plaisant M, Ansari M, Angelidis I, Adler H, Sucre JMS, Taylor CJ, Lin B, Waghray A, Mitsialis V, Dwyer DF, Buchheit KM, Boyce JA, Barrett NA, Laidlaw TM, Carroll SL, Colonna L, Tkachev V, Peterson CW, Yu A, Zheng HB, Gideon HP, Winchell CG, Lin PL, Bingle CD, Snapper SB, Kropski JA, Theis FJ, et al. 2020. SARS-CoV-2 receptor ACE2 is an interferon-stimulated gene in human airway epithelial cells and is detected in specific cell subsets across tissues. Cell 181:1016–1035.e19. doi:10.1016/j.cell.2020.04.035. 32413319 PMC7252096

[33] Yen YT, Liao F, Hsiao CH, Kao CL, Chen YC, Wu-Hsieh BA. 2006. Modeling the early events of severe acute respiratory syndrome coronavirus infection in vitro. J Virol 80:2684–2693. doi:10.1128/JVI.80.6.2684-2693.2006. 16501078 PMC1395447

[34] Van Riel D, Den Bakker MA, Leijten LME, Chutinimitkul S, Munster VJ, De Wit E, Rimmelzwaan GF, Fouchier RAM, Osterhaus ADME, Kuiken T. 2010. Seasonal and pandemic human influenza viruses attach better to human upper respiratory tract epithelium than avian influenza viruses. Am J Pathol 176:1614–1618. doi:10.2353/ajpath.2010.090949. 20167867 PMC2843453

